# Targeted *In Vivo* Inhibition of Specific Protein–Protein Interactions Using Recombinant Antibodies

**DOI:** 10.1371/journal.pone.0109875

**Published:** 2014-10-09

**Authors:** Matej Zábrady, Vendula Hrdinová, Bruno Müller, Udo Conrad, Jan Hejátko, Lubomír Janda

**Affiliations:** 1 Central European Institute of Technology, Masaryk University, Brno, Czech Republic; 2 Institute of Plant Biology & Zürich-Basel Plant Science Center, University of Zürich, Zürich, Switzerland; 3 Department of Molecular Genetics, Leibniz Institute of Plant Genetics and Crop Plant Research (IPK), Gatersleben, Germany; St. Georges University of London, United Kingdom

## Abstract

With the growing availability of genomic sequence information, there is an increasing need for gene function analysis. Antibody-mediated “silencing” represents an intriguing alternative for the precise inhibition of a particular function of biomolecules. Here, we describe a method for selecting recombinant antibodies with a specific purpose in mind, which is to inhibit intrinsic protein–protein interactions in the cytosol of plant cells. Experimental procedures were designed for conveniently evaluating desired properties of recombinant antibodies in consecutive steps. Our selection method was successfully used to develop a recombinant antibody inhibiting the interaction of ARABIDOPSIS HISTIDINE PHOSPHOTRANSFER PROTEIN 3 with such of its upstream interaction partners as the receiver domain of CYTOKININ INDEPENDENT HISTIDINE KINASE 1. The specific down-regulation of the cytokinin signaling pathway *in vivo* demonstrates the validity of our approach. This selection method can serve as a prototype for developing unique recombinant antibodies able to interfere with virtually any biomolecule in the living cell.

## Introduction

The approach of choice for elucidating biological mechanisms is to eliminate the functions of biomolecules and then to observe the responses of the organism. A targeted application of specific inhibitors in order to block a selected biomolecule function allows better control over the process.

Biomolecular interactions constitute the core of biological functions, and these can be defined by their specificity and affinity. Many proteins have evolved into extraordinarily precise biomolecules and can be exploited as specific inhibitors of biomolecular interactions. Antibodies stand out for their unique ability to recognize with relatively high specificity and affinity a virtually unlimited number of target biomolecules, known as antigens. This capability makes antibodies truly indispensable tools in biological research today. Moreover, such tools can be used for antibody-mediated protein “silencing”.

Although the idea to exploit binding properties of recombinant antibodies for *in vivo* applications was proven valid more than two decades ago [1], [2], since that time only a handful of successful examples of intracellularly active antibodies – so-called intrabodies – have been reported. Most such studies conducted on mammalian cells have shown great potential in the treatment of human HIV infection [3], cancer [4], and neurodegenerative diseases [5]. But recombinant antibodies have also found utilization in plant research to induce pathogen resistance, inhibit small molecules, or inhibit function of intrinsic proteins.

Pathogen resistance is an important topic in the biotechnology of plants, because the biological stress induced by pathogens is a major factor influencing agricultural production. Recombinant antibodies have been used for inhibiting viral infections [6], improving resistance to mold [7], and inhibiting bacterial infections [8]. Moreover, plant hormones or their precursors have been successfully inhibited using recombinant antibodies, thereby contributing to deciphering the roles of abscisic acid [9], jasmonate [10], and gibberellin precursors [11] in plant development. Inhibition of other small compounds by recombinant antibodies has also shown potential, such as for inducing herbicide resistance in plants [12]. The recombinant antibodies produced by plants have been termed “plantibodies” and the action of intrabodies is known as “immunomodulation.”

The low redox potential found in the cytosol [13] accounts for difficulties in developing recombinant antibodies active *in vivo* [14], and very few successful such studies in plants have been reported to date [15]–[19]. This scarcity of successful reports points up problems either in targeted selection of recombinant antibodies or relating to the ambiguity of their impact in the living organism [17]. These limitations are being circumvented by, for example, designing novel protein scaffolds with enhanced stability (see review by Grönwall and Ståhl [20]). In order to evaluate the benefits of new protein scaffolds for *in vivo* applications, however, more experimental confirmations need to be produced. Yet another promising approach is to exploit techniques for analyzing protein–protein interactions *in vivo*, and this can be used to streamline the selection process. Several yeast-based hybrid selection systems have been developed [21]–[23], and these were reported, to provide efficient selection of recombinant antibodies along with optimal binding properties for intracellular applications.

In this work, we have designed and verified a novel method for the selection of recombinant antibodies active *in vivo* from diverse sources. This method permits focused development of a specific inhibitor interfering with intrinsic protein–protein interactions in living cells. If the rate of developing candidates for antibody-mediated protein “silencing” will be more efficient, then there will be better availability of these exceptional tools for studying and controlling biological processes.

## Materials and Methods

### Yeast Two-Hybrid Assay

The yeast two-hybrid assay was performed with vectors from Matchmaker System (Clontech). The Gal4 DNA binding domain (BD) protein fusions of AHP proteins have been shown to transactivate the transcription of marker genes in the yeast two-hybrid assay (personal communication J. Horák). Therefore, to eliminate false positive results during the selection process, the pGBKT7 vector, containing the Gal4 DNA binding domain, was used for the cloning of recombinant antibody sequences within NcoI and NotI restriction sites. The pGADT7-DEST vectors encoding AHP proteins fused to Gal4 DNA activation domain (AD) were obtained from Horák et al. [24]. The *S. cerevisiae* strain PJ69-4A (MATa trp1-Δ901 leu2-3,112 901 ura3-52 his3-Δ200 gal4Δ gal8Δ. GAL2-ADE2 LYS2::GAL1-HIS3 met2::GAL7-lacZ) [25] was transformed by the LiAc/PEG method [26] and plated onto transformation selection media composed of a CSM amino acid premix lacking Leu and Trp (MP Biomedicals); 2% glucose; and 6.7 g/L YNB without amino acids (Sigma). The yeast was grown for approximately 3 days and then 5 colonies were inoculated into 0.5 mL of the same liquid media and grown overnight at 30°C. The next day, cultures were diluted to OD_600 nm_ 0.5, grown to OD_600 nm_ 1.0, then plated onto the drop-out media with CSM amino acid premix lacking Leu, Trp and His or Ade. Yeast growth was recorded after 3 days at 30°C. For more stringent selection conditions, the competitive inhibitor of the HIS3 marker gene product 1–5 mM 3-Amino-1,2,4-triazole (Sigma) was added to the media. In addition, liquid yeast cultures were harvested and analyzed by western blot to determine the correct expression of the AHP and recombinant antibody fusion proteins with Gal4 DNA AD and Gal4 DNA BD, respectively (**Figure S1** and **Figure S2**).

### Yeast Three-Hybrid Assay

**Figure 1:**
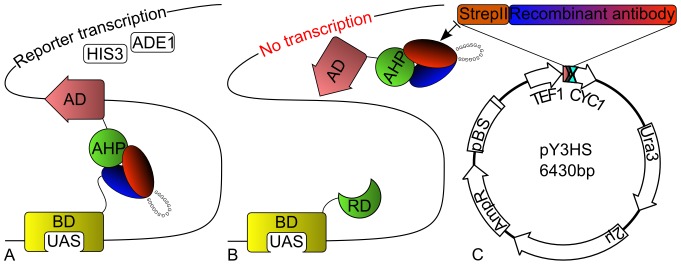
Schematic diagram of yeast two- and three-hybrid assay design with a representative vector map of pY3HS for ectopic expression of the recombinant antibody. (**A**) **Yeast two-hybrid selection.** The interacting partner AHP protein and recombinant antibody fused to the Gal4 AD and BD domains, respectively, allow recruitment of the transcription machinery which activates the expression of nutritional markers HIS3 and ADE1. (**B**) **Yeast three-hybrid selection.** The ectopic expression of recombinant antibody is blocking interaction of the AHP protein and its RD interaction partner. This results in no activation of the expression of nutritional markers HIS3 and ADE1. (**C**) **pY3HS vector map.** Schematic representation of the vector for the ectopic expression of recombinant antibodies in Y3H. (AD =  activation domain, BD =  DNA binding domain, HIS3 =  histidine nutritional marker, ADE1 =  adenine nutritional marker, AHP =  ARABIDOPSIS HISTIDINE PHOSPHOTRANSFER PROTEIN, RD =  ARABIDOPSIS HISTIDINE KINASE receiver domain, TEF1 =  strong constitutive promoter, CYC1 =  terminator, Ura3-uracyl nutritional marker, 2μ =  yeast replication origin, AmpR  =  ampicilin resistance, pBS  =  bacterial replication origin).

The pY3HS vector (**Figure 1C**) is an enhanced version of the pY3H vector (Dualsystems) with the multiple cloning site replaced within the BamHI and XhoI restriction sites, thereby eliminating the BamHI restriction site. The cassette is composed of the BglII restriction site; the N-terminal StrepTag II; the NcoI, NotI, and EcoRI restriction sites; and the stop codon 5′-GATCGAGATCTatgtggtctcacccacaattcgaaaagTCCATGGGCGGCCGCAGAATTCtaaCTCGA-3′. Additionally, the intrinsic NcoI restriction site in the URA3 marker gene sequence of the vector was removed by silent mutation using the QuickChange Lightning Kit (Agilent) with the primer 5′-CTTAACTGTGCCCTCCATcGAAAAATCAGTCAAGATATCC-3′ (silent mutation in lower case). The design of the pY3HS vector allows a simple directional cloning of recombinant antibodies between the NcoI and NotI restriction sites. The pGADT7-DEST vectors encoding AHP proteins and pGBKT7-DEST vector encoding CKI1 RD from Pekárová et al. [27] were co-transformed into the PJ69-4A yeast strain together with pY3HS vector encoding recombinant antibodies. The transformation and handling with the yeast were performed as in the yeast two-hybrid assay, with the exception that the CSM amino acid premix used in the media was additionally lacking Ura. For more stringent selection conditions, the competitive inhibitor of the HIS3 marker gene product 1 mM 3-Amino-1,2,4-triazole (Sigma) was added to the media. In addition, liquid yeast cultures were harvested and analyzed by western blot to determine the correct expression of recombinant antibodies (**Figure S3**).

### Bimolecular Fluorescent Complementation Assay

*A. thaliana* Col-0 plants were grown in a controlled environment on a short day under 100–150 µE light conditions. Bimolecular fluorescent complementation vectors were used for the transfection [28], allowing different combinations of split YFP fusions. The AHP3 [24] without stop codon was sub-cloned by Gateway (Invitrogen) cloning and the scFv-hB7A from pY3HS vector was sub-cloned with the BglII and EcoRI restriction sites. The mesophyll protoplasts were transfected with 10 µg of total DNA following the protocol outlined by Yoo et al. [29], consisting of bimolecular fluorescent complementation vectors with AHP3 and scFv hB7A, together with nuclear-localized mCherry as a transfection control [28]. After 16 h at room temperature to allow maturation of the YFP, the cells were imaged on a Leica SPE confocal scanning light microscope.

### Luciferase Reporter Assay

For the luciferase reporter assay, *A. thaliana* Col-0 plants as well as ahp 1,2,4,5 and ahp 1,3,4,5 mutant lines were grown in a controlled environment on a short day under 100–150 µE light conditions. The ahp 1,2,4,5 and ahp 1,3,4,5 mutant lines were obtained from the ahp 1,2-1,3/+,4,5 line, and genotyping was performed as described previously [30]. Generally, 2×10^4^ mesophyll protoplasts prepared from plants 4–5 weeks old [29] were co-transfected with 20 µg of total DNA, of which 20% consisted of the luciferase reporter TCS:LUC DNA, 20% of the constitutively expressed 35 s:Renilla Luciferase internal control DNA, and including various amounts of the vector DNA containing scFv hB7A (adjusted with pMERM7 – empty vector DNA for control). Each of the three technical replicates of the transfection reaction were split into two samples of 1×10^4^ cells, incubated for 2 h to allow the expression of recombinant antibodies prior to the hormonal treatment, and thereafter one of the samples was treated with 100 nM trans-Zeatin. Each sample was measured independently after 14–16 h of incubation using the Dual-Luciferase Reporter Assay System (Promega).

### Phage Display

All AHP antigens were prepared following the protocol outlined by Pekárová et al. [27]. The protocol for the phage display selection was adopted from Gahrtz and Conrad [31] and performed for the recombinant antibody libraries Human Single Fold scFv Library A+B (hereinafter “scFv library”) [32]. Three rounds of the phage display panning procedure were performed for each of the scFv Libraries with 2 µg/well of the adsorbed AHP3 antigen. From the third round of panning, 96 colonies from each of the scFv libraries were tested. The activity against the AHP3 and the cross-reactivity against the AHP1 protein were simultaneously tested by indirect phage ELISA and by indirect ELISA of soluble recombinant antibodies. The best candidates from each scFv Library were separately tested by indirect ELISA for activity against bovine serum albumin and AHP2, AHP3, AHP5 proteins. The coding sequence of selected specific recombinant antibodies (**List S1**) was determined by sequencing of the respective pIT1 vector DNA and analyzed using CLC Main Workbench (QIAGEN) while following the guidelines published in first parts of the second book of *Antibody Engineering* [33].

### Bacterial Expression of Recombinant Antibodies

Recombinant antibody sequences originating from the phage display phagemid vector pIT1 [31] are bounded by the NcoI and NotI restriction sites. The pET22b(+) (EMD Millipore) bacterial expression vector was used for the expression of recombinant antibodies and was enhanced by a DNA insertion cassette composed of a c-terminal c-myc-tag sequence 5′-GCGGCCGCagaacagaaactgatctctgaagaagacttataaCTCGAG-3′ ending with a stop codon, cloned in between the NotI and XhoI restriction sites. The BL21(DE3) (Invitrogen) bacterial strain was transformed and plated on MDAG-11 media [34] with 200 mg/l ampicillin, and inoculum stocks were prepared from fresh colonies by cultivation in MDAG-11 media with 100 mg/l ampicillin at 250 rpm and 37°C for 8 h. After centrifugation at 1000 rpm, the bacterial pellet was frozen in 10% glycerol in 1/10 of the initial culture volume. TBM-5052 was used for the expression. This essentially is ZYM-5052 auto-induction media [34] with 12 g/l tryptone and 24 g/l yeast extract instead of the ZY and trace metals from the M9 minimal media recipe [35]. Erlenmeyer flasks filled to 5% of the maximum volume were inoculated with 1/100 of the inoculum stock and cultivated at 250 rpm and 22°C for 24 h. To prepare periplasmic extracts, the harvested bacterial culture was resuspended in 5 ml/1 g wet cell weight of the extraction buffer (200 mM Boric acid, 150 mM NaCl, 1 mM EDTA, pH 8) and incubated on a vertical shaker for 3 h at 4°C. The periplasmic extract was the recovered supernatant fraction after centrifugation at 10 000 rcf for 30 minutes. The *in vitro* activity of recombinant antibodies produced in bacteria was tested by far-western blot. The total protein extracts of the bacterial expression of six AHP proteins were prepared following the protocol outlined by Pekárová et al. [27], separated by SDS-PAGE, electroblotted onto a PVDF membrane in Towbin buffer (25 mM Tris-HCl, 150 mM glycine, 10% methanol, pH 8.3) and blocked for 1 h in blocking buffer (5% skimmed milk, TBS, 0.1% Tween-20). The amount of the total protein extract loaded on SDS-PAGE gels were normalized for each of the AHP protein by comparing the detection of incorporated 6x-His-tag with 1∶5000 primary anti-6x-His-tag antibody (Sigma) and the 1∶20 000 secondary anti-mouse IgG AP conjugated antibody (Sigma). The periplasmic extract containing recombinant antibodies was diluted 1∶2 in the blocking buffer and applied for 1 h, and the bound recombinant antibody was detected with 1∶50 primary anti-c-myc-tag antibody (hybridoma line 50 supernatant) and the 1∶10 000 secondary anti-mouse IgG AP conjugated antibody (Sigma).

### ELISA

The periplasmic extract of recombinant antibodies from 4 L culture prepared as described before was precipitated overnight in 60% ammonium sulfate at 4°C and the precipitate was recovered after 20 000 rcf for 30 minutes. The precipitated extract was reconstituted in 30 mL Protein L equilibration buffer and loaded onto a 1 mL Protein L column (Pierce) and purified following manufacturer instructions. The purified and concentrated recombinant antibody in PBS was evaluated for purity on SDS-PAGE gel.

The specificity of recombinant antibodies was determined as followed. Purified AHP1, AHP2, AHP3 and AHP5 were coated in (50 mM bi-carbonate buffer, pH 9.6) overnight onto Maxisorp plates (Nunc), 500 ng/well at 4°C. The following steps were performed for 1 h at 37°C with agitation. Wells were blocked in the blocking buffer (5% skimmed milk, PBS, 0.05% Tween-20) and recombinant antibodies in different concentrations. Bound recombinant antibodies were detected with 1∶50 primary anti-c-myc-tag antibody (hybridoma line 50 supernatant) and 1∶10 000 secondary anti-mouse IgG HRP conjugated antibody (Sigma) with TMB substrate (TestLine).

The affinity of scFv hB7A was measured in competitive ELISA. The 20 nM AHP3 antigen was coated and blocked as described earlier. The following steps were performed for 1 h at 37°C with agitation. The 45 nM scFv hB7A was equilibrated with 2-fold dilutions of 1950 nM AHP3 in 500 µL PBS buffer. For each dilution, four 100 µL samples were added to AHP3 coated wells. Bound recombinant antibodies were detected with 1∶10 primary anti-c-myc-tag antibody (hybridoma line 50 supernatant) and 1∶200 secondary anti-mouse IgG AP conjugated antibody (Sigma) with pNPP substrate. The mean and standard deviation of absorbance units collected from two independent measurements were fitted with DYNAFIT software [36] for dissociation constant.

## Results

### The Yeast Two-Hybrid Assay is an Efficient Technique for Screening the Activity of Recombinant Antibodies in vivo

**Figure 2:**
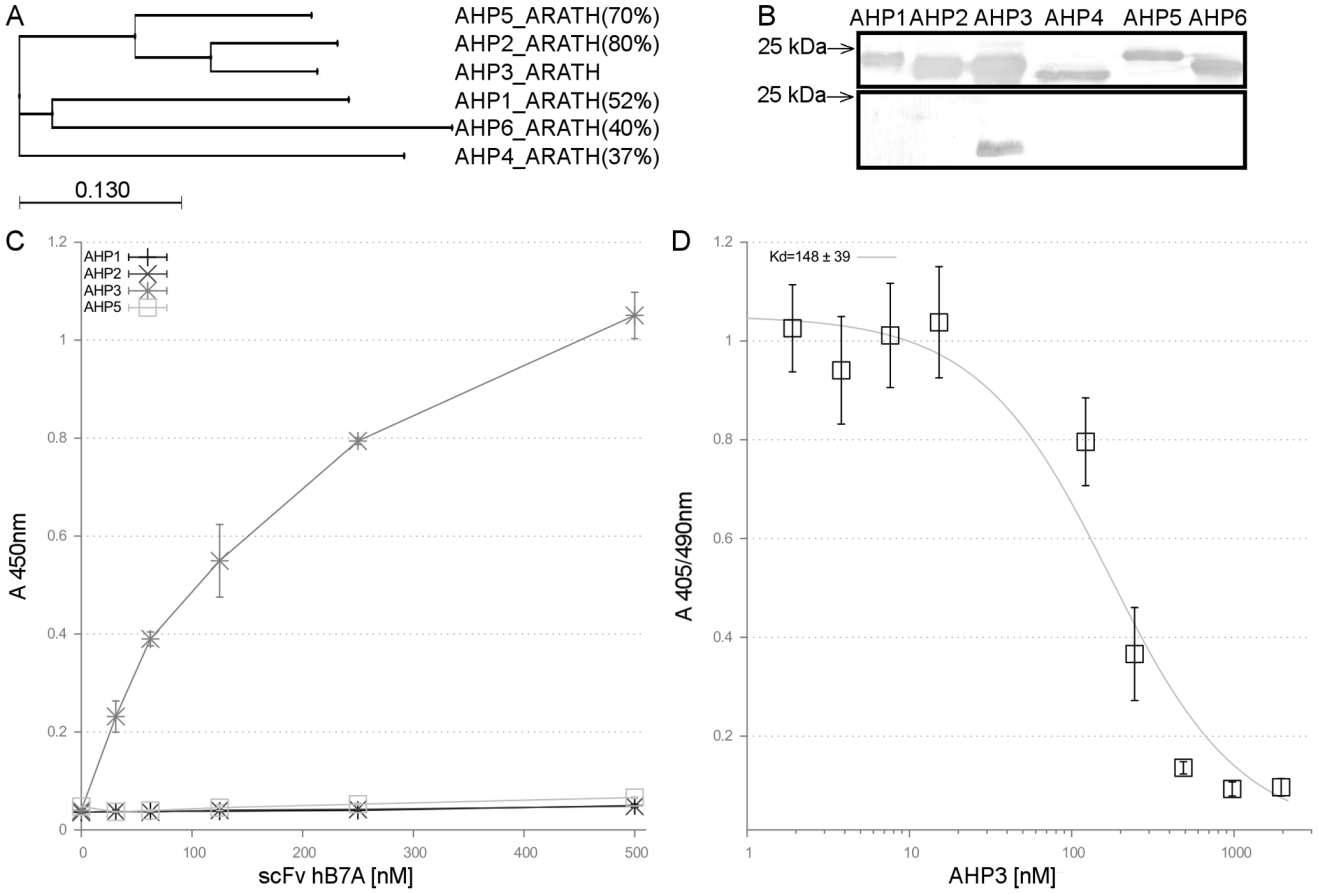
*In vitro* characterization of scFv hB7A to AHP antigens. (**A**) **Sequence variability of AHP proteins amino acid sequence.** ClustalW tree of ARABIDOPSIS HISTIDINE PHOSPHOTRANSFER PROTEINS (AHP1-6). Amino acid sequence identity of AHP proteins (compared to AHP3 in brackets). (**B**) **Far-western blot of recombinant AHP proteins.** Recombinant protein expression with incorporated 6x-His-tag detected was confirmed by anti-6x-His-tag antibody immunodetection (top); AHP proteins were detected by recombinant scFv hB7A from the periplasmic extract of the bacterial expression, incorporated c-myc-tag detected by anti-c-myc-tag antibody (bottom). (**C**) **The specificity of scFv hB7A against AHP proteins tested in indirect ELISA.** Absorbance values of triplicates (±SD represented with error bars) at 450 nm are displayed for each AHP protein (500 ng/well). (**D**) **The affinity of scFv hB7A to AHP3 tested in competitive ELISA.** Absorbance values at 405 nm normalized to 490 nm of quadruplicates from two independent measurements were pooled (±SD represented with error bars). The AHP3 was coated at 20 nM and 2-fold dilutions of 1950 nM AHP3 was equilibrated with 45 nM scFv hB7A prior loading to wells. The data was fitted with DYNAFIT software.

Careful consideration was given to choosing the most appropriate technique for screening the *in vivo* activity of recombinant antibodies. The yeast two-hybrid assay is a widely used technique for evaluating protein–protein interactions, and this concept was adopted (**Figure 1A**). Recombinant antibodies were tested in this assay against all six highly homologous AHP proteins (**Figure 2A**).

**Figure 3:**
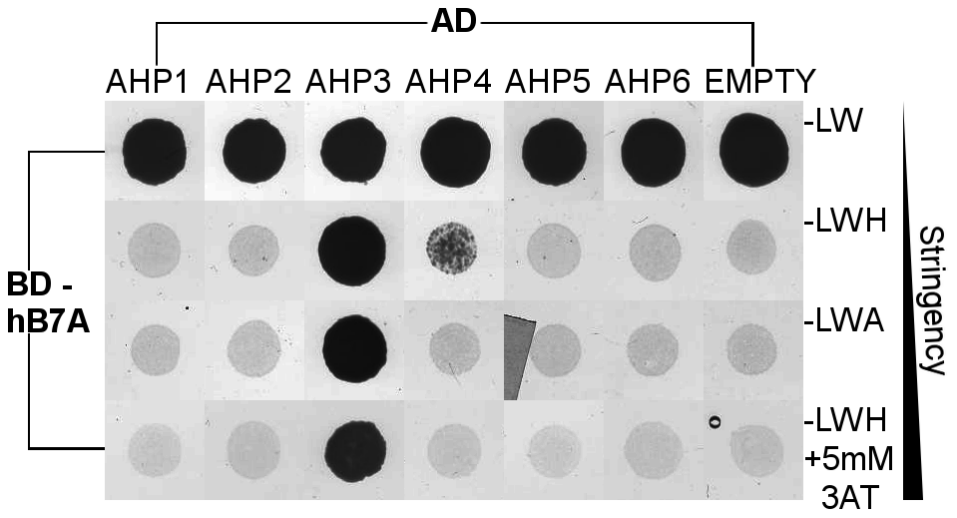
*In vivo* binding specificity of scFv hB7A. The scFv hB7A (Gal4 DNA binding domain BD fusion) and six AHP proteins (Gal4 activation domain AD fusion) in the yeast two-hybrid assay. The visible yeast growth was recorded after incubation for 3 days on a different yeast drop-out media lacking Leu (L), Trp (W) and His (H) or Ade (A). For more stringent conditions 5 mM 3-Amino-1,2,4-triazole (3AT) was added to the media. The empty pGATD7 was used as negative control. The interaction of scFv hB7A with AHP3 is represented by a visible yeast growth under stringent conditions.

The scFv hB7A specifically interacted only with AHP3, thus corresponding with its *in vitro* activity (**Figure 2B** and **Figure 2C**). The yeast growth endured in the more stringent selection conditions of 5 mM 3-Amino-1,2,4-triazole (**Figure 3**), thus suggesting a strong interaction between AHP3 and scFv hB7A (**Figure 2D**).

While other recombinant antibodies did not interact specifically with any of the AHP proteins, the data obtained demonstrate that the yeast two-hybrid assay is an effective technique for the first step in the selection method (**Table S1**). The ability to screen large DNA libraries by means of yeast two-hybrid assay will be useful for setting up a high-throughput screening step.

### The Ectopic Expression scFv hB7A in Yeast Three-Hybrid Assay Inhibits Protein–Protein Interactions

The aim of this selection method was to develop a potent recombinant antibody for the antibody-mediated protein “silencing”. Therefore, to ascertain the ability of the selected recombinant antibody to inhibit a specific protein–protein interaction *in vivo*, a yeast three-hybrid assay was adopted (**Figure 1B**). The scFv hB7A in pY3HS for the ectopic expression was co-transformed together with CKI1 RD and its natural interaction partners AHP2, AHP3 and AHP5 in respective vectors.

**Figure 4:**
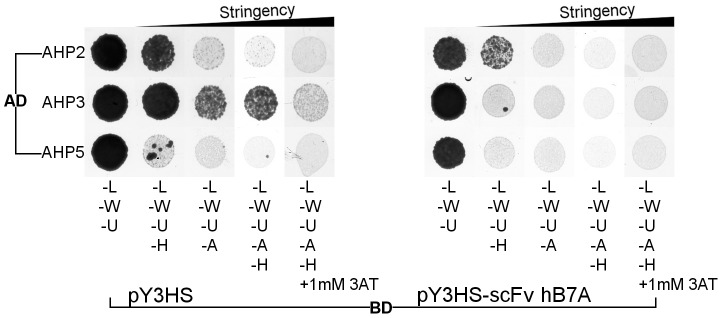
*In vivo* inhibition of AHP3-CKI1 RD interaction. CKI1 receiver domain (RD) (Gal4 DNA binding domain BD fusion) interaction with respective AHP interaction partners (Gal4 activation domain AD fusion) upon ectopic expression of scFv hB7A. The visible yeast growth was recorded after incubation for 3 days on a different yeast drop-out media lacking Leu (L), Trp (W), Ura (U) and His (H) or Ade (A) or with added 1 mM 3-Amino-1,2,4-triazole (3AT). Compared to the empty vector pY3HS, scFv hB7A specifically inhibits interactions of AHP3 in the yeast three-hybrid assay as indicated by visible growth inhibition of yeast in different drop-out media. The interaction of AHP5 is naturally very weak and so it is difficult to observe in the yeast three-hybrid assay.

With the ectopic expression of scFv hB7A, the recorded yeast growth was inhibited only for the interaction partners AHP3 – CKI1 RD, while other interaction partners of CKI1 RD were not significantly affected (**Figure 4**).

CKI1 RD is upstream from the AHP proteins in the signaling pathway. Therefore, other upstream interaction partners of the AHP3 were tested and similar inhibitory effects of scFv hB7A *in vivo* were shown. The design of this verification step allows for complex evaluation of the inhibitory effects of selected recombinant antibodies within a single experiment.

### The scFv hB7A Interacts with AHP3 in A. thaliana

Before undertaking laborious experiments to elucidate the effect of the scFv hB7A on the cytokinin signaling pathway, a last verification step was designed to determine its activity in the cytosol of *A. thaliana*. The interaction of scFv hB7A with the AHP3 was confirmed by bimolecular fluorescent complementation assay [28].

**Figure 5:**
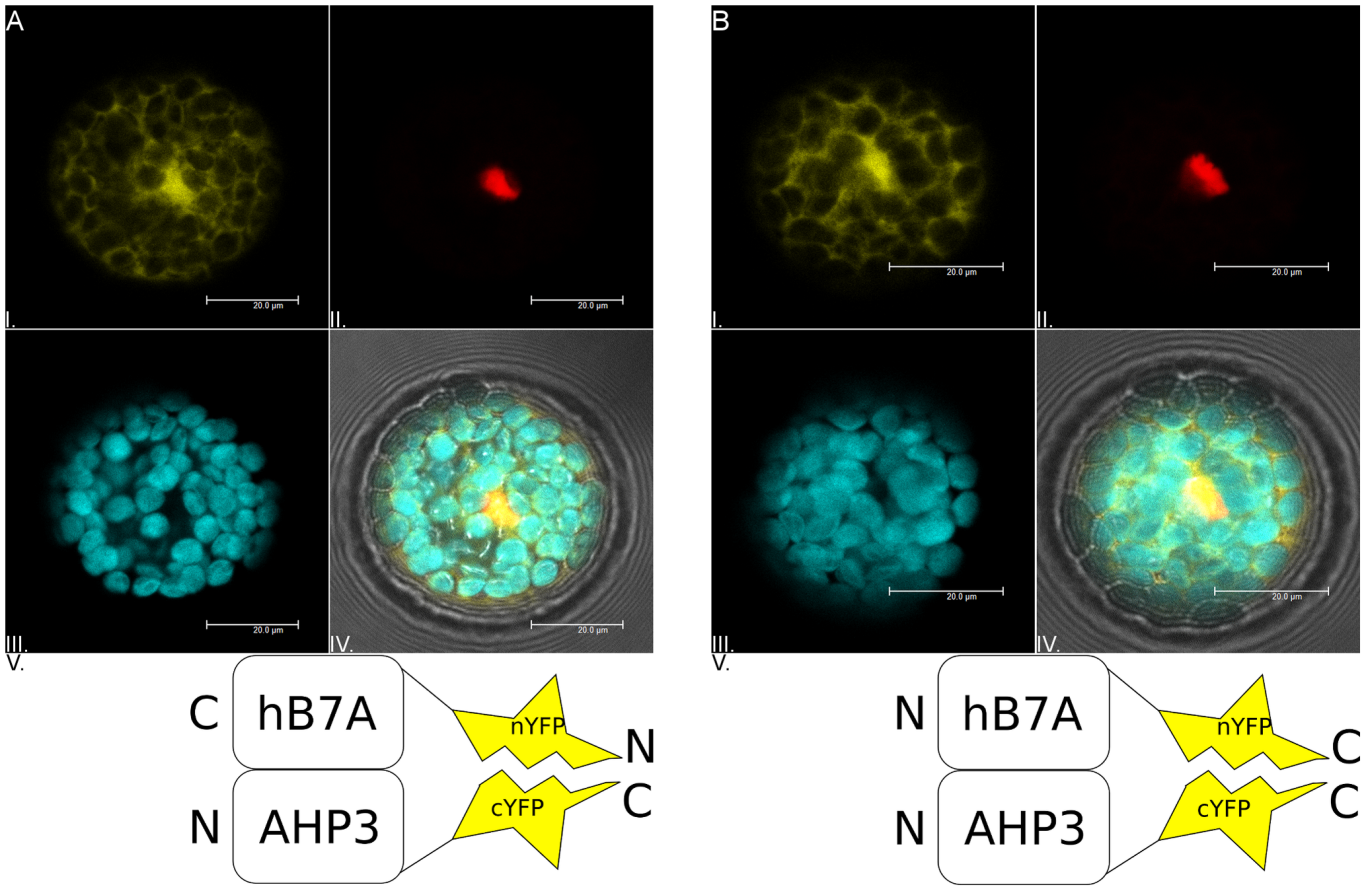
Recombinant antibody scFv hB7A is interacting with AHP 3 in *A. thaliana*. Confocal images of *A. thaliana* mesophyll protoplasts co-transformed with nYFP:scFv-hB7A (**A**) and scFv-hB7A:nYFP (**B**) interacts irrespective of the fusion orientation with AHP3:cYFP (I. - yellow channel). The signal of the reconstituted split-YFP protein is localized in the cytosol and in the nucleus. Nuclear localized mCherry (II. - red channel) and autofluorescence (>650 nm) of chloroplasts (III. - cyan channel) serve as co-localization markers. The integrity of the cell is visible from the overlay picture together with transmission channel (IV.). Schematic representation of the experiment (V.) Negative control is shown in (**Figure S8**). Scale bars: 20 µm.

The interaction was visualized while the shorter C-terminal fragment of the YFP was fused to the C-terminal part of the AHP3. The fusion orientation of the longer N-terminal fragment of YFP with the scFv hB7A was not influencing the interaction (**Figure 5**). These results were interpreted, that the binding epitope of scFv hB7A is at the N-terminal part or in the central part of the AHP3. The epitope mapping performed with the trypsin-digested AHP3 indicates, the binding epitope is close to the phosphorylation site of H82 (**Figure S4**).

This observation might not be in agreement with the results obtained from the yeast two-hybrid assay, where both AHP3 and scFv hB7A are C-terminal protein fusions. The bioinformatic analysis showed that the AHP3 in the yeast two-hybrid assay forms a longer peptide linker with the adjacent Gal4 AD protein, and therefore scFv hB7A has a better accessibility to bind AHP3.

### The scFv hB7A–Mediated Protein Silencing of AHP3 Down–Regulates the Cytokinin Signaling Pathway in A. thaliana

It was important to quantify the inhibitory effects of scFv hB7A (selected in consecutive steps as described above) on the cytokinin signaling pathway. The information about potency of the selected inhibitor is valuable for the design of future experiments involving stable transgenic plants ectopically expressing the aforementioned recombinant antibody. With the help of unique techniques based on the TEAMP [29] and TCS:LUC reporter assay [37], the response of the cytokinin signaling pathway upon transient expression of scFv hB7A in *A. thaliana* mesophyll protoplasts was measured.

Because the functional redundancy of endogenous AHP1-5 proteins was predicted to mask the AHP3 specific effect of scFv hB7A on the cytokinin signaling pathway [38], quadruple ahp 1,2,4,5 mutant plants were used and compared with ahp 1,3,4,5 mutant plants along with Col-0 wild type plants as controls. The relative increase of luminescence between the untreated and 100 nM trans-Zeatin treated samples defined the activity of the cytokinin signaling pathway.

**Figure 6:**
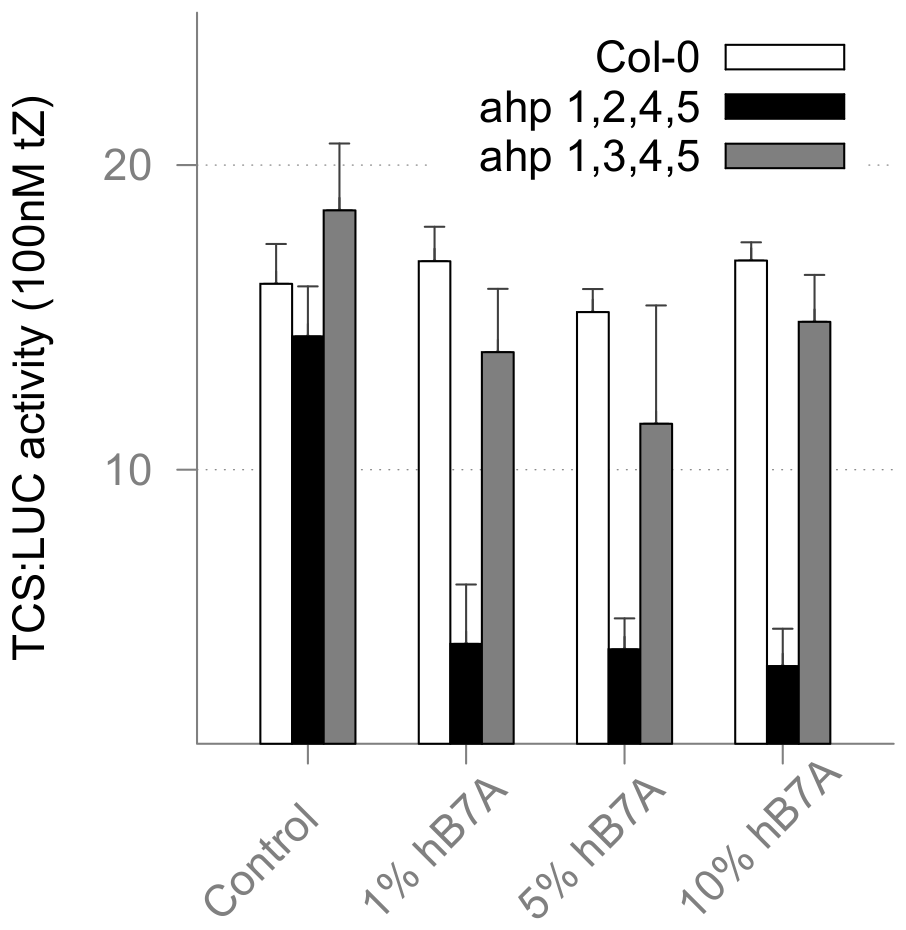
Specific down-regulation of the cytokinin signaling pathway in *A. thaliana* mesophyll protoplasts after ectopic expression of scFv hB7A. Levels of the TCS:LUC induced luciferase activity correspond to the activity of the cytokinin signaling pathway activated with 100 nM trans-Zeatin (tZ) and normalized to the transfection control 35S:Renilla Luciferase. The ectopic expression of scFv hB7A is sorted by the amount of total (20 µg) co-transfected DNA (expressed in percent). The Col-0 wild type plants (white) and ahp 1,3,4,5 mutant plants with solitary active AHP2 (gray) show no significant effect. The ahp 1,2,4,5 mutant plants (black) with solitary active AHP3 were severely affected

The ectopic expression of scFv hB7A did not affect the cytokinin signaling pathway activity of Col-0 wild type plants and ahp 1,3,4,5 mutant plants. The levels of cytokinin signaling pathway were strongly compromised in the ahp 1,2,4,5 mutant plants (**Figure 6**), thus showing the targeted inhibition of endogenous AHP3.

The reduced relative amount of the vector DNA encoding scFv hB7A used for the transformation was still able dramatically to inhibit the cytokinin signaling pathway. This result can be interpreted as showing that scFv hB7A is a very potent inhibitor of AHP3 and is specifically interfering with AHP3 function within the cytokinin signaling pathway. Additionally, the AHP3 “node” was not removed from the complex protein–protein interaction network and adverse effects resulting from such a removal were thereby minimized.

## Discussion

In this article, we report the first successful demonstration of recombinant antibodies inhibiting intrinsic protein–protein interactions in the cytosol of plants. The selection method used to achieve antibody-mediated protein “silencing” is composed of four consecutive steps. Each step is carefully designed to contribute to successful selection of the *in vivo* active recombinant antibody which is specifically inhibiting protein–protein interactions, and its impact after the ectopic expression can be measured directly in *A. thaliana*.

To provide proof of concept for the study's findings, ARABIDOPSIS HISTIDINE PHOSPHOTRANSFER PROTEINS (AHP) were chosen as the target for inhibition. AHP1-5 proteins comprise five redundant and highly homologous components of the cytokinin signaling pathway [38], which is a complex regulatory network in *A. thaliana* involving promiscuous protein–protein interactions [39]. They act through multi-step phosphorylation, transmitting the perceived signal from membrane-bound receptors to the nucleus. The inhibition of underlying interactions of AHP proteins within the multi-step phosphorylation will specifically disable the cytokinin signaling pathway.

Numerous recombinant antibodies with different *in vitro* specificity against AHP proteins (**Table S1**.) were selected prior to actual screening for *in vivo* activity (**Figure S5** and **Figure S6**). Selection and characterization of AHP protein-specific single domain recombinant antibodies (sdAb) [40] is under study with the aim of exploring this additional source.

In the first step, yeast two-hybrid assay was chosen as the most appropriate technique. It had previously been used successfully to confirm interactions of the components of the *A. thaliana* cytokinin signaling pathway [27]. From all tested recombinant antibodies, only scFv hB7A was found active *in vivo*. Interestingly, another three recombinant antibodies selected from the same source as scFv hB7A (hA6H, hA11C, hB3H) were not found to be active, although they are composed of an identical amino acid sequence in the framework regions (**Figure S7**). Therefore, the discovery of a recombinant antibody framework with inherited *in vivo* activity [41] was not possible in this study.

The purpose of the yeast three-hybrid assay in the next step was to evaluate the inhibition of protein–protein interactions *in vivo*. The scFv hB7A showed itself to be a competitive inhibitor of AHP3 – CKI1 RD interaction. The pY3HS vector used in this step is compatible with other yeast two-hybrid assays and can be quickly adopted for other pre-existing experiments.

The bimolecular fluorescent complementation assay shed more light as to where the potential binding epitope of scFv hB7A is located. The C-terminal fusion of YFP protein to AHP3 hindered the binding of scFv hB7A, thus indicating that the binding epitope is on the other part of the protein. The recombinant antibody remained active, irrespective of the split YFP protein fusion orientation. This might allow functional protein fusions of scFv hB7A in order to enhance or control its inhibitory activity.

The final step of the method designed in this study was directed to measuring the impact of scFv hB7A-mediated protein “silencing” of AHP3. This was accomplished using a synthetic promoter-driven expression of a luciferase reporter which reflects activation of the cytokinin signaling pathway [37]. Designed to measure hormonal responses and benefiting from the sensitivity of the luminescent enzymatic reporter, TEAMP and TCS:LUC provided a precise and sensitive technique for evaluating recombinant antibodies *in planta*. The data from experiments performed show that scFv hB7A is a highly potent and specific inhibitor of AHP3 interaction with such of its upstream interaction partners as CKI1 RD.

This technique is not limited only to the cytokinin signaling pathway. Other reporters have been developed for plants, such as the auxin responsive element known as DR5 [42]. Thus, virtually any promoter sequence can be used to drive the expression of the luciferase reporter. Because the inhibition of any biomolecule affecting the activity of a given promoter can be conveniently measured, this method can be applied for a broad range of target molecules.

The antibody-mediated protein “silencing,” or immunomodulation, approach used in this study is greatly limited by the uncertainty of successfully selecting good candidates. The benefits are nevertheless substantial and very well exemplified in success stories regarding proteins that are difficult to study. The complete genetic elimination of ABP1 protein has been found lethal in the embryonic stage of development [43], and only the complementary approach of RNA silencing and the specific recombinant antibody [44] has enabled its role in development of *A. thaliana* to be deciphered [45]–[47].

To summarize, we have established a new strategy that allows targeted selection of specific and potent inhibitory recombinant antibodies. The four steps in the selection method are fast and reliable, and a successful *de novo* selection of *in vivo* active recombinant antibodies might be anticipated within a reasonable period. The selection method was verified in a case study demonstrating specific inhibition of the cytokinin signaling pathway. The scFv hB7A-mediated AHP3 “silencing” provides a tool for more precisely examining the role of AHP3 in *A. thaliana*. In selecting scFv hB7A, and with the evidence of its activity, we demonstrate that our method can be used for selecting unique *in vivo* active recombinant antibodies with desired functions. This method is modular and highly adaptable for many experimental needs, and any source of recombinant antibodies or other binding scaffolds can be compatible with its use.

## Supporting Information

Figure S1 **An example western blot of AHP1-6 protein fusions with Gal4 AD from the yeast two-hybrid assay.** Lanes 1. and 9. - AHP1, lanes 2. and 10. - AHP2, lanes 3. and 11. - AHP3, lanes 4. and 12. - AHP4, lanes 5. and 13. - AHP5, lanes 6. and 14. - AHP6, lanes 7. and 15. – Control protein, 8. - PageRuler Prestained Protein Ladder 10–170 K (Pierce).
(TIFF)

Figure S2 **An example western blot of the recombinant antibody protein fusions with Gal4 DNA BD from the yeast two-hybrid assay.** Lanes 1.-7.- scFv hB7A, lane 8. - PageRuler Prestained Protein Ladder 10–170 K (Pierce), lanes 9.-15. - scFv m1A10.
(TIFF)

Figure S3 **An example western blot of the ectopic expression of scFv hB7A from the yeast three-hybrid assay.** Lanes 1. and 20. - PageRuler Prestained Protein Ladder 10-170 K (Pierce), lanes 2.-4. CKI1 RD interactions with AHP proteins, lane 5. scFv hB7A interaction with AHP3 protein, lanes 6.-10. ETR1 RD interactions with AHP proteins, lanes 11.-15. AHK5 RD interactions with AHP proteins, lanes 16.-19. in AHK4 interactions with AHP proteins.
(TIFF)

Figure S4 **The MALDI-TOF analysis of the binding epitope of scFv hB7A from AHP3.** (**A**) **The comparison of identified AHP3 peptides between negative control and sample**. Negative control is 50 µg AHP3 protein, 1∶50 digested with trypsin and inhibited with 1 mM PMSF after 30 minutes. The digest was loaded and eluted from 1 mL Protein L column (Pierce). The sample was prepared like the negative control, with 50 µg of scFv hB7A added to the reaction after PMSF inhibition, to enrich the elution fraction with specific peptides. (**B**) **The aminoacid sequence of identified peptides with their theoretical molecular weight**. (**C**) **Homology model of AHP3 based on PDB 4G78** (54.9% sequence identity). The identified peptides are highlighted green with His82 shown in red.
(TIFF)

Figure S5 **The specificity of scFv hA11C against AHP proteins tested in indirect ELISA.** Absorbance values of triplicates (±SD represented with error bars) at 450 nm are displayed for each AHP protein (500 ng/well).
(TIFF)

Figure S6 **The specificity of scFv hA6H against AHP proteins tested in indirect ELISA.** Absorbance values of triplicates (±SD represented with error bars) at 450 nm are displayed for each AHP protein (500 ng/well).
(TIFF)

Figure S7 **A simple protein alignment of scFv hB7A, hA11C, hA6H and hB3H.** Identical aminoacids are represented with dots (.) and the annotated framework regions (FR) are shown.
(TIF)

Figure S8 **Recombinant antibody scFv hB7A is not interacting with CKI1 RD in** ***A. thaliana*** **.** A negative control for confocal images of *A. thaliana* mesophyll protoplasts co-transformed with nYFP:scFv-hB7A and cYFP:CKI1 RD (I. - yellow channel). Nuclear localized mCherry (II. - red channel) and autofluorescence (>650 nm) of chloroplasts (III. - cyan channel) serve as co-localization markers. The integrity of the cell is visible from the overlay picture together with transmission channel (IV.). Schematic representation of the experiment (V.) Scale bars: 20 µm.
(TIFF)

Table S1 **A table of recombinant antibodies tested for the activity against AHP proteins, categorized by used techniques.** The definition of symbols: (+) positive, (−) negative, (N/A) not determined.
(PDF)

List S1 **DNA sequences of recombinant antibodies selected from Human Single Fold scFv Library A + B.**
(PDF)

Methods S1 **Western Blots of Yeast Extracts.**
(DOCX)
