# Supplementary figures and images for: Targeted In Vivo Inhibition of Specific Protein–Protein Interactions Using Recombinant Antibodies
Source: PLoS One. 2014 Oct 9;9(10):e109875. doi: 10.1371/journal.pone.0109875 (PMC4192540; doi:10.1371/journal.pone.0109875)

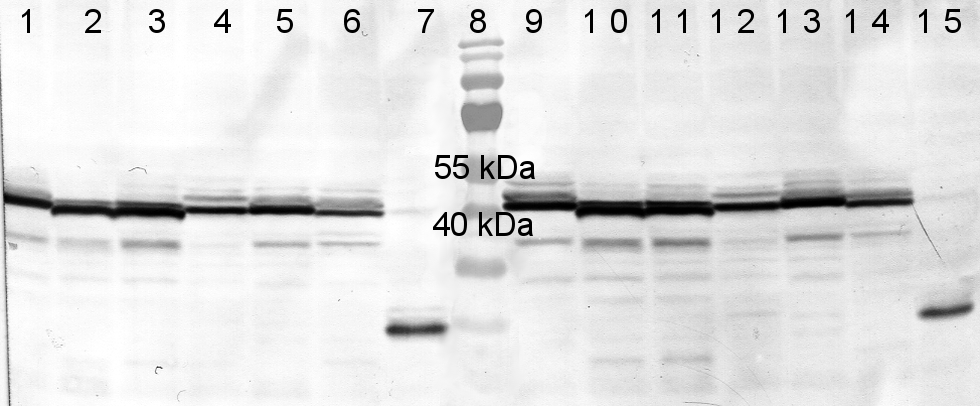

Supplement: Figure S1 — An example western blot of AHP1-6 protein fusions with Gal4 AD from the yeast two-hybrid assay. Lanes 1. and 9. - AHP1, lanes 2. and 10. - AHP2, lanes 3. and 11. - AHP3, lanes 4. and 12. - AHP4, lanes 5. and 13. - AHP5, lanes 6. and 14. - AHP6, lanes 7. and 15. – Control protein, 8. - PageRuler Prestained Protein Ladder 10–170 K (Pierce). (TIFF) [file pone.0109875.s001.tiff]

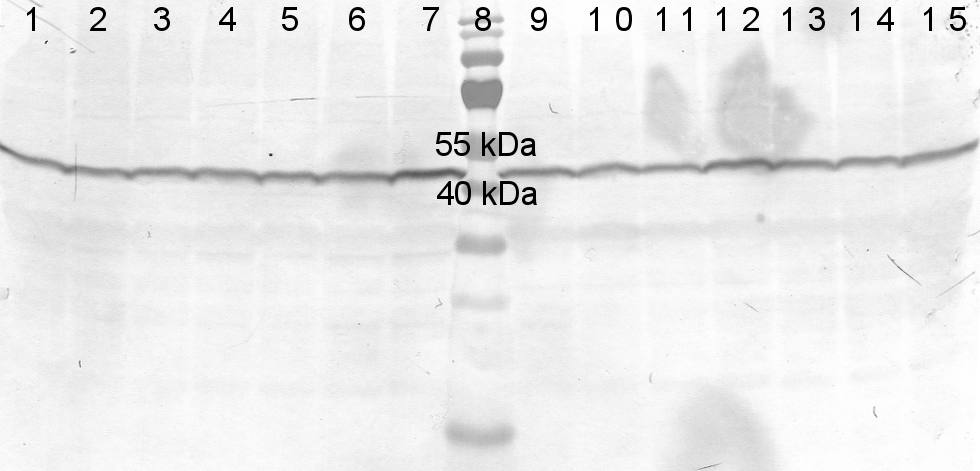

Supplement: Figure S2 — An example western blot of the recombinant antibody protein fusions with Gal4 DNA BD from the yeast two-hybrid assay. Lanes 1.-7.- scFv hB7A, lane 8. - PageRuler Prestained Protein Ladder 10–170 K (Pierce), lanes 9.-15. - scFv m1A10. (TIFF) [file pone.0109875.s002.tiff]

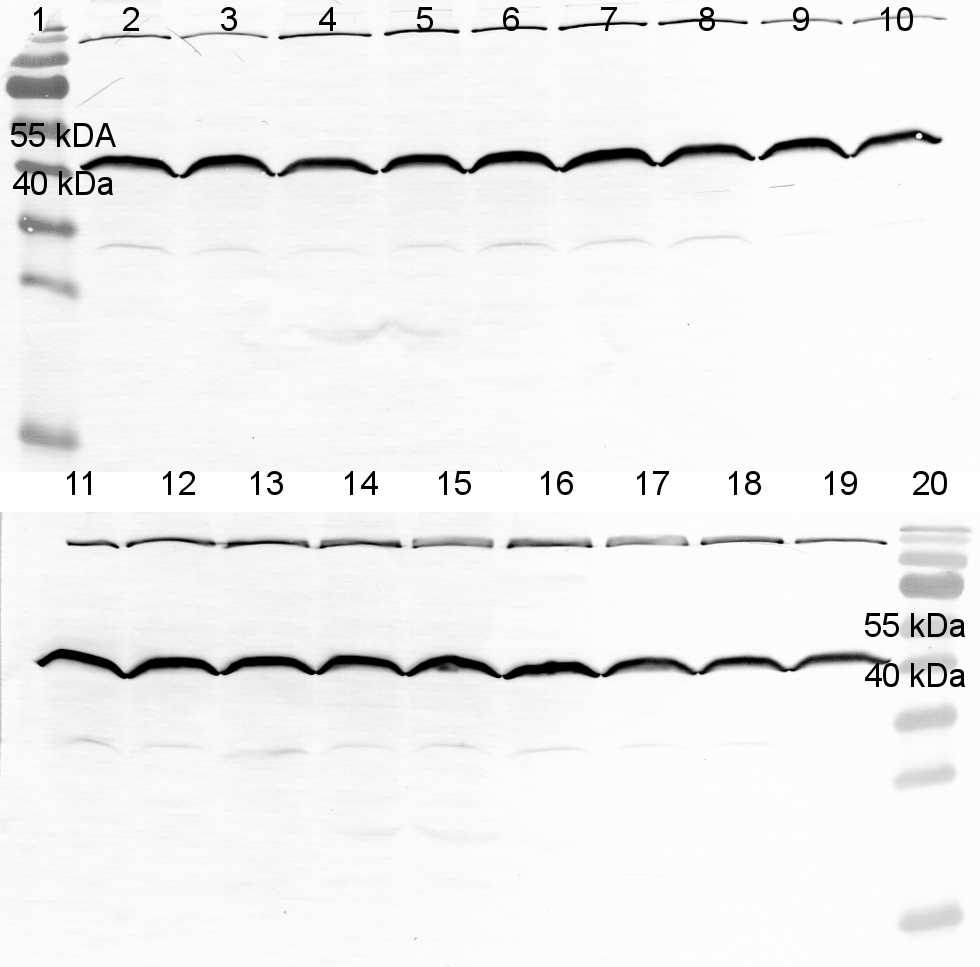

Supplement: Figure S3 — An example western blot of the ectopic expression of scFv hB7A from the yeast three-hybrid assay. Lanes 1. and 20. - PageRuler Prestained Protein Ladder 10-170 K (Pierce), lanes 2.-4. CKI1 RD interactions with AHP proteins, lane 5. scFv hB7A interaction with AHP3 protein, lanes 6.-10. ETR1 RD interactions with AHP proteins, lanes 11.-15. AHK5 RD interactions with AHP proteins, lanes 16.-19. in AHK4 interactions with AHP proteins. (TIFF) [file pone.0109875.s003.tiff]

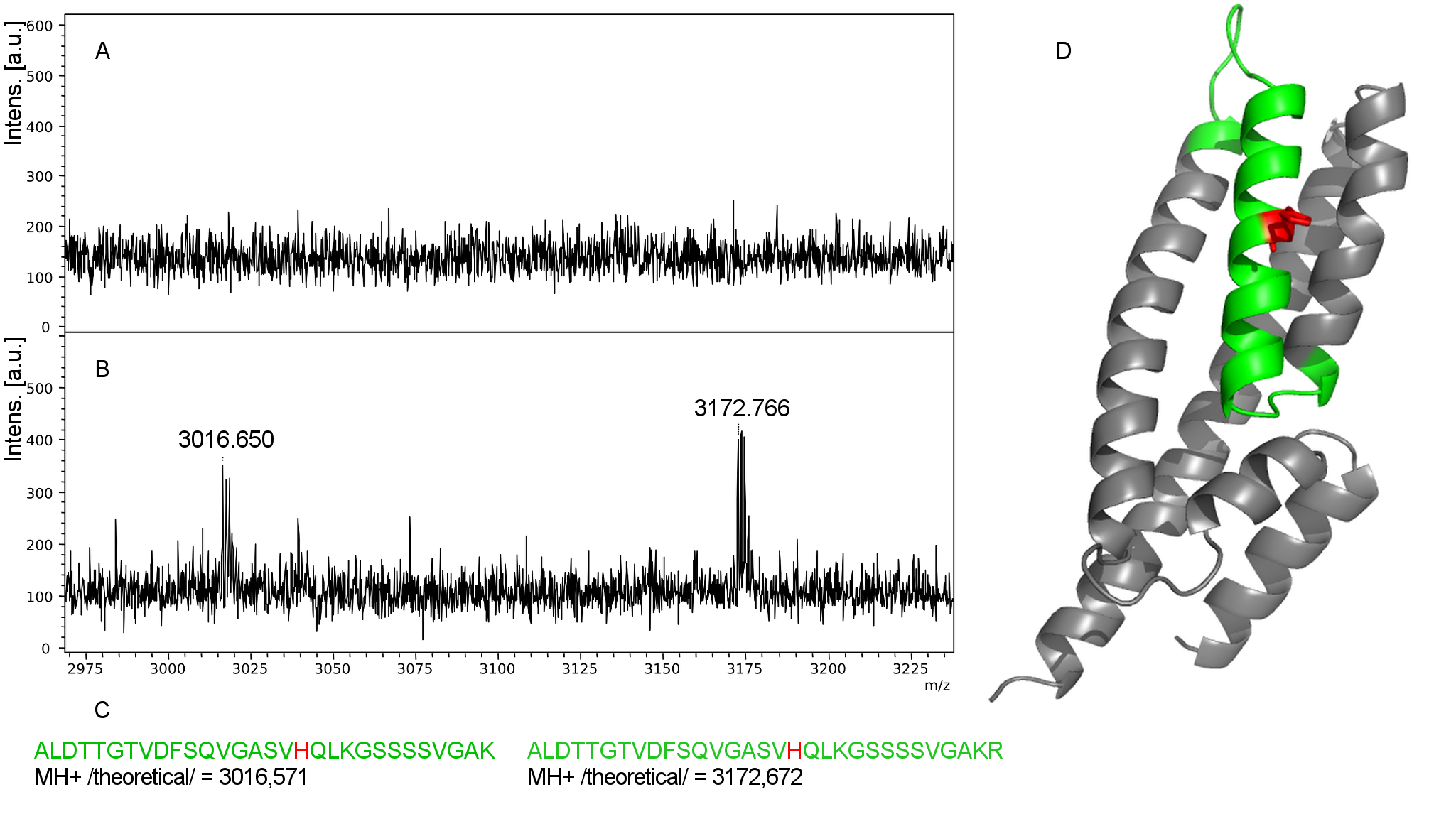

Supplement: Figure S4 — The MALDI-TOF analysis of the binding epitope of scFv hB7A from AHP3. (A) The comparison of identified AHP3 peptides between negative control and sample. Negative control is 50 µg AHP3 protein, 1∶50 digested with trypsin and inhibited with 1 mM PMSF after 30 minutes. The digest was loaded and eluted from 1 mL Protein L column (Pierce). The sample was prepared like the negative control, with 50 µg of scFv hB7A added to the reaction after PMSF inhibition, to enrich the elution fraction with specific peptides. (B) The aminoacid sequence of identified peptides with their theoretical molecular weight. (C) Homology model of AHP3 based on PDB 4G78 (54.9% sequence identity). The identified peptides are highlighted green with His82 shown in red. (TIFF) [file pone.0109875.s004.tiff]

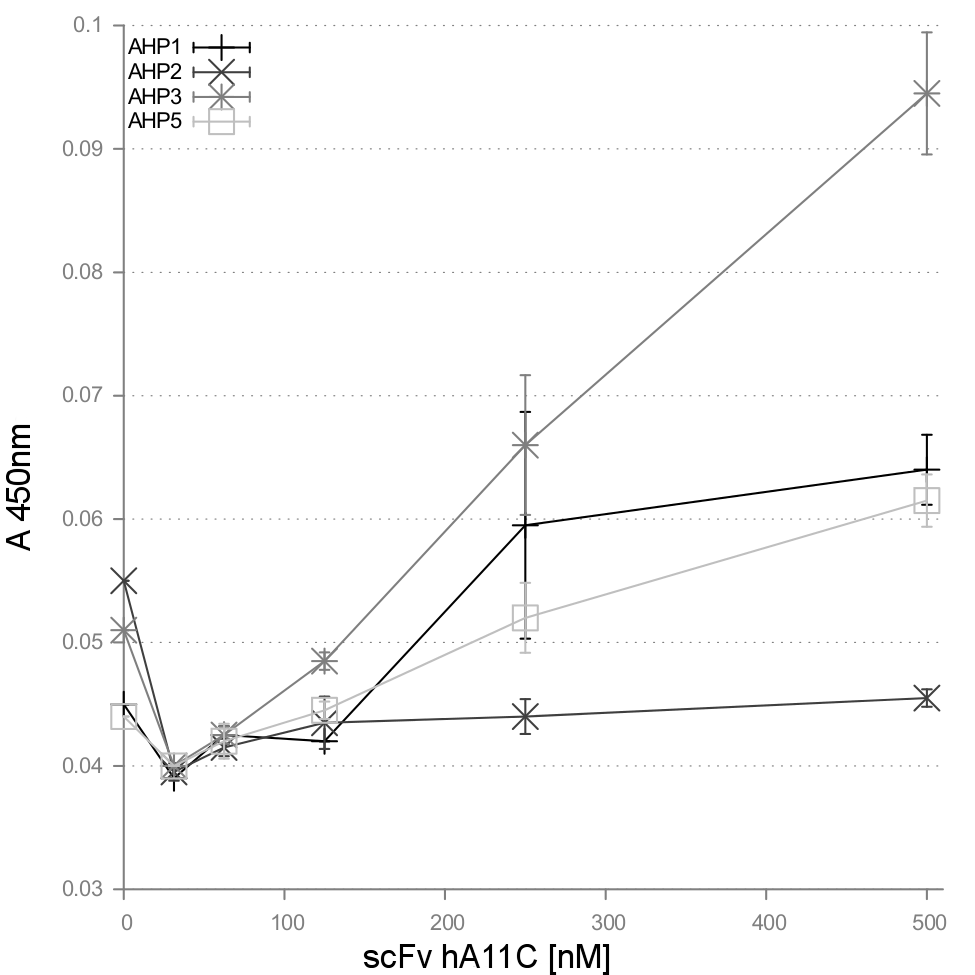

Supplement: Figure S5 — The specificity of scFv hA11C against AHP proteins tested in indirect ELISA. Absorbance values of triplicates (±SD represented with error bars) at 450 nm are displayed for each AHP protein (500 ng/well). (TIFF) [file pone.0109875.s005.tiff]

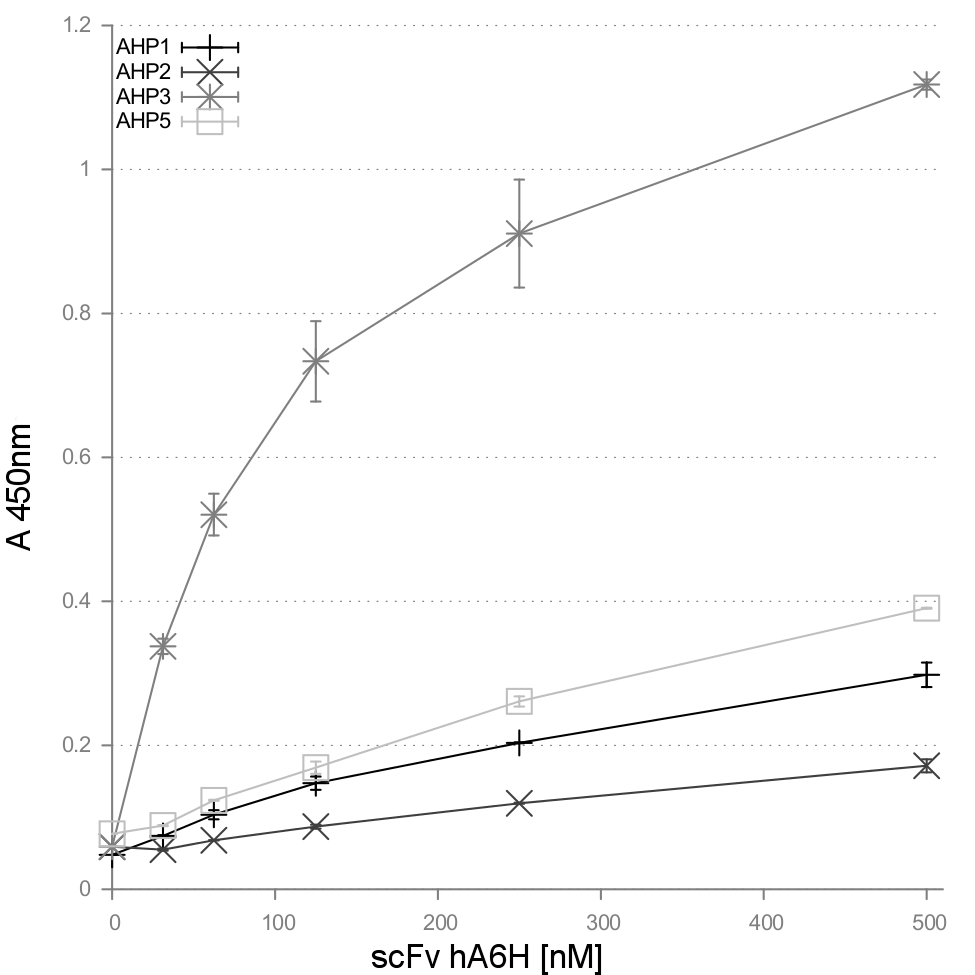

Supplement: Figure S6 — The specificity of scFv hA6H against AHP proteins tested in indirect ELISA. Absorbance values of triplicates (±SD represented with error bars) at 450 nm are displayed for each AHP protein (500 ng/well). (TIFF) [file pone.0109875.s006.tiff]

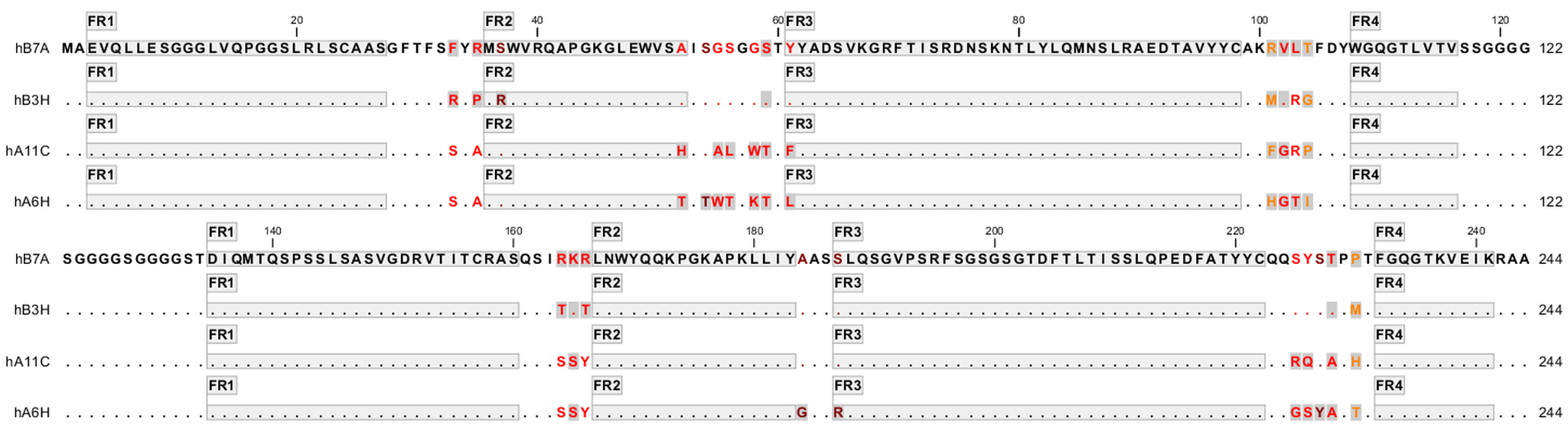

Supplement: Figure S7 — A simple protein alignment of scFv hB7A, hA11C, hA6H and hB3H. Identical aminoacids are represented with dots (.) and the annotated framework regions (FR) are shown. (TIF) [file pone.0109875.s007.tif]

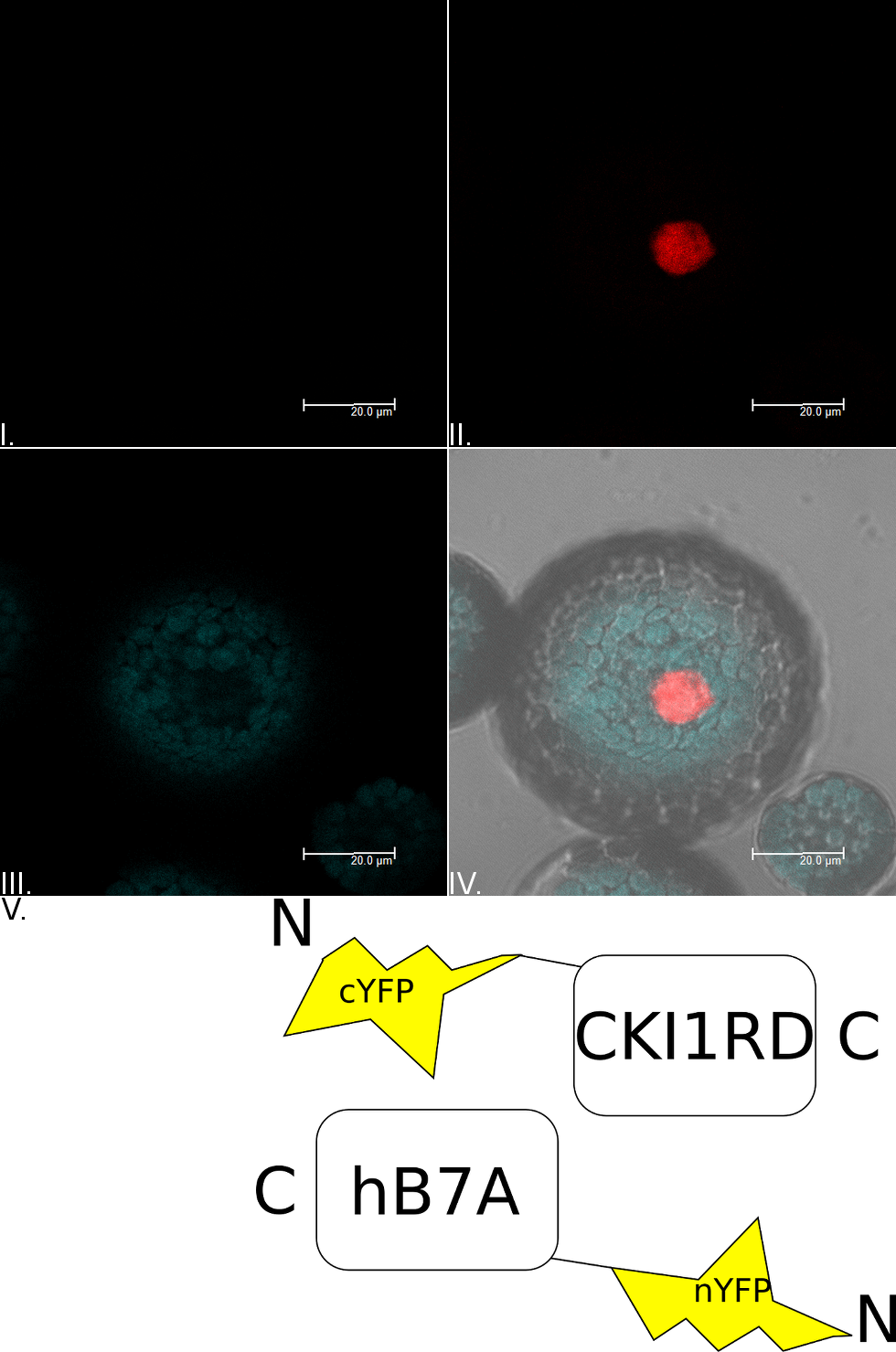

Supplement: Figure S8 — Recombinant antibody scFv hB7A is not interacting with CKI1 RD in A. thaliana . A negative control for confocal images of A. thaliana mesophyll protoplasts co-transformed with nYFP:scFv-hB7A and cYFP:CKI1 RD (I. - yellow channel). Nuclear localized mCherry (II. - red channel) and autofluorescence (>650 nm) of chloroplasts (III. - cyan channel) serve as co-localization markers. The integrity of the cell is visible from the overlay picture together with transmission channel (IV.). Schematic representation of the experiment (V.) Scale bars: 20 µm. (TIFF) [file pone.0109875.s008.tiff]
