# Supplementary material for: Targeted In Vivo Inhibition of Specific Protein–Protein Interactions Using Recombinant Antibodies
Source: PLoS One. 2014 Oct 9;9(10):e109875. doi: 10.1371/journal.pone.0109875 (PMC4192540; doi:10.1371/journal.pone.0109875)
Supplement: Table S1 — A table of recombinant antibodies tested for the activity against AHP proteins, categorized by used techniques. The definition of symbols: (+) positive, (−) negative, (N/A) not determined. (PDF) [file pone.0109875.s009.pdf]

ELISA	AHP1	AHP2	AHP3	AHP4	AHP5	AHP6	Control
scFv hA6H	+++	++	+++	N/A	+++	N/A	-
scFv hA11C	-	-	+/-	N/A	+/-	N/A	-
scFv hB7A	-	-	+++	N/A	-	N/A	-
scFv hB3H	+/-	+/-	+/-	N/A	+/-	N/A	+
scFv 1A10	N/A	N/A	N/A	N/A	N/A	N/A	N/A
scFv 5B10	N/A	N/A	N/A	N/A	N/A	N/A	N/A
scFv 2	N/A	N/A	N/A	N/A	N/A	N/A	N/A
scFv 13	N/A	N/A	N/A	N/A	N/A	N/A	N/A
scFv A4	N/A	N/A	N/A	N/A	N/A	N/A	N/A
scFv A3	N/A	N/A	N/A	N/A	N/A	N/A	N/A

WB	AHP1	AHP2	AHP3	AHP4	AHP5	AHP6	Control
scFv hA6H	+	-	+	N/A	+	N/A	N/A
scFv hA11C	N/A	N/A	N/A	N/A	N/A	N/A	N/A
scFv hB7A	-	-	+	-	-	-	N/A
scFv hB3H	N/A	N/A	N/A	N/A	N/A	N/A	N/A
scFv 1A10	-	-	+	-	-	-	N/A
scFv 5B10	-	-	-	-	-	-	N/A
scFv 2	-	-	-	-	-	-	N/A
scFv 13	N/A	N/A	N/A	N/A	N/A	N/A	N/A
scFv A4	-	-	-	-	-	-	N/A
scFv A3	-	-	-	-	-	-	N/A

Y2H	AHP1	AHP2	AHP3	AHP4	AHP5	AHP6	Control
scFv hA6H	-	-	-	-	-	-	N/A
scFv hA11C	-	-	-	-	-	-	N/A
scFv hB7A	-	-	+	-	-	-	N/A
scFv hB3H	-	-	-	-	-	-	N/A
scFv 1A10	-	-	-	+/-	-	-	N/A
scFv 5B10	-	-	-	-	-	-	N/A
scFv 2	-	-	-	-	-	-	N/A
scFv 13	-	-	-	-	-	-	N/A
scFv A4	-	-	-	-	-	-	N/A
scFv A3	-	-	-	-	-	-	N/A
