# Supplementary material for: Targeted In Vivo Inhibition of Specific Protein–Protein Interactions Using Recombinant Antibodies
Source: PLoS One. 2014 Oct 9;9(10):e109875. doi: 10.1371/journal.pone.0109875 (PMC4192540; doi:10.1371/journal.pone.0109875)
Supplement: List S1 — DNA sequences of recombinant antibodies selected from Human Single Fold scFv Library A + B. (PDF) [file pone.0109875.s010.pdf]

>hA6H Anti AHP3 Ab
CCATGGCCGAGGTGCAGCTGTTGGAGTCTGGGGGAGGCTTGGTACAGCCTGGGGGGTCCCTGAGACTCTCCTGTG
CAGCCTCTGGATTCACCTTTAGCAGCTATGCCATGAGCTGGGTCCGCCAGGCTCCAGGGAAGGGGCTGGAGTGGG
TCTCAACTATTACTTGGACGGGTAAGACGACACTTTACGCAGACTCCGTGAAGGGCCGGTTCACCATCTCCAGAG
ACAATTCCAAGAACACGCTGTATCTGCAAATGAACAGCCTGAGAGCCGAGGACACGGCCGTATATTACTGTGCGA
AACATGGTACTATTTTTGACTACTGGGGCCAGGGAACCCTGGTCACCGTCTCGAGCGGTGGAGGCGGTTCAGGCG
GAGGTGGCAGCGGCGGTGGCGGGTCGACGGACATCCAGATGACCCAGTCTCCATCCTCCCTGTCTGCATCTGTAG
GAGACAGAGTCACCATCACTTGCCGGGCAAGTCAGAGCATTAGCAGCTATTTAAATTGGTATCAGCAGAAACCAG
GGAAAGCCCCTAAGCTCCTGATCTATGGTGCATCCAGGTTGCAAAGTGGGGTCCCATCAAGGTTCAGTGGCAGTG
GATCTGGGACAGATTTCACTCTCACCATCAGCAGTCTGCAACCTGAAGATTTTGCAACTTACTACTGTCAACAGG
GGTCTTATGCGCCTACTACGTTCGGCCAAGGGACCAAGGTGGAAATCAAACGGGCGGCCGC

>hA11C Anti AHP3 Ab
CCATGGCCGAGGTGCAGCTGTTGGAGTCTGGGGGAGGCTTGGTACAGCCTGGGGGGTCCCTGAGACTCTCCTGTG
CAGCCTCTGGATTCACCTTTAGCAGCTATGCCATGAGCTGGGTCCGCCAGGCTCCAGGGAAGGGGCTGGAGTGGG
TCTCACATATTTCTGCTCTTGGTTGGACTACATTTTACGCAGACTCCGTGAAGGGCAGGTTCACCATCTCCAGAG
ACAATTCCAAGAACACGCTGTATCTGCAAATGAACAGCCTGAGAGCCGAGGACACGGCCGTATATTACTGTGCGA
AATTTGGGCGGCCTTTTGACTACTGGGGCCAGGGAACCCTGGTCACCGTCTCGAGCGGTGGAGGCGGTTCAGGCG
GAGGTGGCAGCGGCGGTGGCGGGTCGACGGACATCCAGATGACCCAGTCTCCATCCTCCCTGTCTGCATCTGTAG
GAGACAGAGTCACCATCACTTGCCGGGCAAGTCAGAGCATTAGCAGCTATTTAAATTGGTATCAGCAGAAACCAG
GGAAAGCCCCTAAGCTCCTGATCTATGCTGCATCCAGTTTGCAAAGTGGGGTCCCATCAAGGTTCAGTGGCAGTG
GATCTGGGACAGATTTCACTCTCACCATCAGCAGTCTGCAACCTGAAGATTTTGCAACTTACTACTGTCAACAGC
GGCAGAGTGCTCCTCATACGTTCGGCCAAGGGACCAAGGTGGAAATCAAACGGGCGGCCGC

>hB3H Anti AHP3 Ab
CCATGGCCGAGGTGCAGCTGTTGGAGTCTGGGGGAGGCTTGGTACAGCCTGGGGGGTCCCTGAGACTCTCCTGTG
CAGCCTCTGGATTCACCTTTAGCAGGTATCCTATGCGTTGGGTCCGCCAGGCTCCAGGGAAGGGGCTGGAGTGGG
TCTCAGCTATTAGTGGTAGTGGTGGTAGCACATACTACGCAGACTCCGTGAAGGGCCGGTTCACCATCTCCAGAG
ACAATTCCAAGAACACGCTGTATCTGCAAATGAACAGCCTGAGAGCCGAGGACACGGCCGTATATTACTGTGCGA
AAATGGTTCGGGGGTTTGACTACTGGGGCCAGGGAACCCTGGTCACCGTCTCGAGCGGTGGAGGCGGTTCAGGCG
GAGGTGGCAGCGGCGGTGGCGGGTCGACGGACATCCAGATGACCCAGTCTCCATCCTCCCTGTCTGCATCTGTAG
GAGACAGAGTCACCATCACTTGCCGGGCAAGTCAGAGCATTACTAAGACTTTAAATTGGTATCAGCAGAAACCAG
GGAAAGCCCCTAAGCTCCTGATCTATGCTGCATCCAGTTTGCAAAGTGGGGTCCCATCAAGGTTCAGTGGCAGTG
GATCTGGGACAGATTTCACTCTCACCATCAGCAGTCTGCAACCTGAAGATTTTGCAACTTACTACTGTCAACAGA
GTTACAGTACCCCTATGACGTTCGGCCAAGGGACCAAGGTGGAAATCAAACGGGCGGCCGC

>hB7A Anti AHP3 Ab
CCATGGCCGAGGTGCAGCTGTTGGAGTCTGGGGGAGGCTTGGTACAGCCTGGGGGGTCCCTGAGACTCTCCTGTG
CAGCCTCTGGATTCACCTTTAGCTTTTATAGGATGTCGTGGGTCCGCCAGGCTCCAGGGAAGGGGCTGGAGTGGG
TCTCAGCTATTAGTGGTAGTGGTGGTAGCACATACTACGCAGACTCCGTGAAGGGCCGGTTCACCATCTCCAGAG
ACAATTCCAAGAACACGCTGTATCTGCAAATGAACAGCCTGAGAGCCGAGGACACGGCCGTATATTACTGTGCGA
AACGGGTGCTTACGTTTGACTACTGGGGCCAGGGAACCCTGGTCACCGTCTCGAGCGGTGGAGGCGGTTCAGGCG
GAGGTGGCAGCGGCGGTGGCGGGTCGACGGACATCCAGATGACCCAGTCTCCATCCTCCCTGTCTGCATCTGTAG
GAGACAGAGTCACCATCACTTGCCGGGCAAGTCAGAGCATTCGTAAGCGTTTAAATTGGTATCAGCAGAAACCAG
GGAAAGCCCCTAAGCTCCTGATCTATGCTGCATCCAGTTTGCAAAGTGGGGTCCCATCAAGGTTCAGTGGCAGTG
GATCTGGGACAGATTTCACTCTCACCATCAGCAGTCTGCAACCTGAAGATTTTGCAACTTACTACTGTCAACAGA
GTTACAGTACCCCTCCTACGTTCGGCCAAGGGACCAAGGTGGAAATCAAACGGGCGGCCGC
