# Supplementary material for: Targeted In Vivo Inhibition of Specific Protein–Protein Interactions Using Recombinant Antibodies
Source: PLoS One. 2014 Oct 9;9(10):e109875. doi: 10.1371/journal.pone.0109875 (PMC4192540; doi:10.1371/journal.pone.0109875)
Supplement: Methods S1 — Western Blots of Yeast Extracts. (DOCX) [file pone.0109875.s011.docx]

**Supporting Information Methods**

***Western Blots of Yeast Extracts***

Yeast cultures (2 mL) from drop-out tests in the yeast two- and yeast-three hybrid assay were peleted at OD_600 nm_ 1.0 at 4000 rcf for 1 minute. The cell pellet was resuspended in 100 μL water and 100 μL 0.2 M NaOH was added for 5 minutes at room temperature. Samples were peleted at 4000 rcf for 1 minute and the supernatant was aspirated. The cell pellet was resuspended in 33 μL of 1x Laemmli loading buffer and denatured at 95°C for 10 minutes . Samples were centrifuged for 1 minute at 20 000 rcf and 15 μL were loaded on a 15% discontinuous SDS-PAGE gels. Separated proteins were electroblotted onto a PVDF membrane in Towbin buffer for 1 h at 100V and blocked in the blocking buffer (5% skimmed milk, TBS, 0.1% Tween-20). Gal4 AD protein fusion (HA-tag) was detected with 1:10 000 primary anti-HA-tag antibody (Roche) and 1:20 000 secondary anti-rat IgG AP conjugated antibody (Sigma). Gal4 DNA BD protein fusions (c-myc-tag) with 1:200 primary anti-c-myc-tag antibody (hybridoma line 50 supernatant) and 1:20 000 secondary anti-mouse IgG AP conjugated antibody (Sigma). The ectopic expression of recombinant antibodies in the yeast three-hybrid assay, was detected with 1:5000 Strep-Tactin AP conjugated (IBA).
